# Increased plasma brain-derived neurotrophic factor (BDNF) as a potential biomarker for and compensatory mechanism in mild cognitive impairment: a case-control study

**DOI:** 10.18632/aging.203598

**Published:** 2021-10-15

**Authors:** Ted Kheng Siang Ng, Christina Coughlan, Patricia C. Heyn, Alex Tagawa, James J. Carollo, Ee Heok Kua, Rathi Mahendran

**Affiliations:** 1 Department of Psychological Medicine, Yong Loo Lin School of Medicine, National University of Singapore, Singapore 119228, Singapore; 2 University of Colorado Anschutz Medical Campus, Aurora, CO 80045, USA; 3 Arizona State University, Edson College of Nursing and Health Innovation, Phoenix, AZ 85004, USA; 4 National Cheng Kung University, Institute of Behavioral Medicine, College of Medicine, Tainan 701, Taiwan; 5 University of Colorado Alzheimer’s and Cognition Center (CUACC), Department of Neurology, School of Medicine, University of Colorado Anschutz Medical Campus, Aurora, CO 80045, USA; 6 Children’s Hospital Colorado, Center for Gait and Movement Analysis (CGMA), Aurora, CO 80045, USA; 7 Department of Psychological Medicine, National University Hospital, Singapore 119228, Singapore

**Keywords:** BDNF, hs-CRP, discriminative accuracy, mild cognitive impairment, cerebral palsy

## Abstract

Background: Previous meta-analyses examining the continuum of Alzheimer’s disease (AD) concluded significantly decreased peripheral brain-derived neurotrophic factor (BDNF) in AD. However, across different meta-analyses, there remain inconsistent findings on peripheral BDNF levels in individuals with mild cognitive impairment (MCI). This issue has been attributed to the highly heterogenous clinical and laboratory factors. Thus, BDNF’s level, discriminative accuracy for identifying all-cause MCI and its subtypes, and its associations with other biomarkers and neurocognitive domains, remain largely unknown.

Methods: To address this heterogeneity, we compared a healthy control cohort (n=56, 45 female) to an MCI cohort (n=40, 28 female), to determine whether plasma BDNF, hs-CRP, and DHEA-S can differentiate healthy from MCI individuals, including two MCI subtypes (amnestic [aMCI] and non-amnestic [non-aMCI]). The associations between BDNF with other biomarkers and neurocognitive tests were examined. Adults with cerebral palsy were included as sensitivity analyses.

Results: Compared to healthy controls, BDNF was significantly higher in all-cause MCI, aMCI, and non-aMCI. Furthermore, BDNF had good (AUC=0.84, 95% CI=0.74 to 0.95, p<0.001) and excellent discriminative accuracies (AUC=0.92, 95% CI=0.84 to 1.00, p<0.001) for all-cause MCI and non-amnestic MCI, respectively. BDNF was significantly and positively associated with plasma hs-CRP (β=0.26, 95% CI=0.02 to 0.50, p=0.038), despite attenuated association upon controlling for BMI (β=0.15, 95% CI=-0.08 to 0.38, p=0.186). Multiple inverse associations between BDNF and detailed neurocognitive tests were also detected.

Conclusions: These findings suggest BDNF is increased as a compensatory mechanism in preclinical dementia, supporting the neurotrophic and partially the inflammatory hypotheses of cognitive impairment.

## INTRODUCTION

Mild cognitive impairment (MCI) is an intermediate state between normal aging and early dementia [1–3]. Individuals with MCI have an increased risk of dementia and can be broadly categorised into two subtypes with distinct clinical trajectories; amnestic-MCI (a-MCI) cases, which typically progress into Alzheimer’s disease (AD), while the majority of cases with non-amnestic-MCI (non-aMCI) develop non-Alzheimer’s dementia [2]. Regardless of subtypes, MCI is a clinical diagnosis, made primarily based on the established gold standard Peterson’s criteria [2], which relies on clinical judgement informed by reviewing participants’ self-reported cognitive complaints and neurocognitive test scores. Thus, clinicians often disagree on a diagnosis of MCI, with the Cronbach alpha value for an MCI diagnosis often only slightly higher than 0.5. Hence, an objective biomarker is much needed, particularly one which also illuminates the biological underpinnings of MCI. A biomarker has several advantages in screening and triaging a clinical diagnosis; in addition to being less time-consuming than administering a comprehensive battery of neurocognitive tests, it serves as an objective measure free from the influence of interviewer and patient bias. Biomarkers have been increasingly recognized as an important component to guide precision medicine [4], which has the goal of identifying, characterizing, and personalizing effectively, screening, diagnosis and treatments that are unique to the needs of an individual patient [4]. As such, biomarkers could assist clinicians in more accurately differentiating those with MCI from healthy aging older adults in the screening process. As MCI represents an optimal stage for initiating disease-modifying interventions, with improved screening and diagnostic accuracies, clinically-useful biomarkers would allow interventions being delivered in a more timely and targeted manner.

Biomarkers are also imperative in understanding the biological underpinnings of MCI. The neuropathologies associated with cognitive impairment (CI) and dementia are complex, multi-faceted, and inter-related. Neuropathological hallmarks of Alzheimer’s dementia include beta-amyloid (Aβ) deposition, tau hyperphosphorylation, and neurodegeneration [5, 6]. The amyloid/tau/neurodegeneration (A/T/N) framework [7] has thus been proposed. Several AD biomarkers, including cerebrospinal fluid (CSF) [8] and positron emission tomography (PET) imaging of amyloid and tau proteins [9, 10], have been extensively validated and show high sensitivities and specificities, but their levels do not provide the desired information with respect to staging the disease process. This lack of staging where someone is in the neurodegenerative disease process results primarily from a lack of consistent cut-off values for Aβ, Tau etc. that determine when someone moves from for e.g. MCI to AD, a deficiency that needs to be urgently addressed. Several research groups have shown non-Aβ, non-tau (NANT) markers as candidates for neurodegeneration, with Brain-derived neurotropic factor (BDNF) serving as one example [11–15]. BDNF is a neurotrophin that promotes the survival, functions, and development of neurons [16]. BDNF also modulates cognition and memory, by promoting neurogenesis and synaptic growth, enhancing neurotransmission across synapses, and modulating synaptic plasticity [17]. It is also involved in inducing hippocampal long-term potentiation, an essential mechanism for memory formation [17]. BDNF is widely expressed in the brain, including cortex, hippocampus, and the basal forebrain regions. It crosses the blood-brain-barrier in a bidirectional manner and is thus detectable in the blood [18]. Weinstein et al. found that older adults with higher peripheral BDNF levels had lower odds of developing AD [19]. BDNF level was also altered in many other neurodegenerative diseases and psychiatric disorders [20–23]. Decreased BDNF levels may thus constitute a lack of trophic support, contributing to neuronal degeneration [24]. Hence, BDNF could be a candidate biomarker for neurodegeneration in MCI. We have previously conducted a meta-analysis and concluded that peripheral BDNF is significantly decreased in patients with AD, compared to healthy controls [25]. Other meta-analyses show similar and consistent findings [26, 27]. However, no consensus had been reached regarding peripheral BDNF levels in MCI. Primary studies and even meta-analyses often present inconsistent and contradictory evidence on the levels of peripheral BDNF in MCI. One of the main explanations for such discrepant findings is the presence of significant and high heterogeneity across studies [25, 28]. Furthermore, it was proposed that MCI represents an early stage in the trajectory of dementia, where peripheral BDNF levels may be increased as a compensatory and neuroprotective strategy in response to various neuronal insults [14, 15, 19, 27, 29]. This hypothesis is further supported by studies reporting increased peripheral BDNF levels in MCI [14, 15, 30].

Two main sources of heterogeneity present in previous peripheral BDNF studies are clinical factors and laboratory measures. Amongst the critical clinical factors is the high heterogeneity in older adults presenting with MCI, many having psychiatric co-morbidities [25], such as major depressive disorder (MDD) and generalized anxiety disorder (GAD) [31, 32], complicating the diagnosis. Since patients with MDD and GAD have decreased peripheral BDNF levels [23, 33], the presence of these psychiatric co-morbidities may mask the real changes in peripheral BDNF in MCI. The consumption of psychotropic medications is another prominent source of heterogeneity in accurately assessing peripheral BDNF levels. Most peripheral BDNF studies recruited participants with MCI from clinical settings, who were consuming various psychotropic medications. Some of these medications, such as anti-depressants, have been reported to restore peripheral BDNF to normal levels in patients with MDD [23, 34] making this a major confounder in accurately measuring baseline BDNF levels. Furthermore, most of the extant studies were conducted in the Western hemisphere, with a lower representation of MCI cases in individuals of Asian ethnicity.

Different lab approaches have also been noted to cause high heterogeneity in the peripheral measurements of BDNF. Different sample types (plasma versus serum) used to quantify peripheral BDNF could provide vastly different insights. Platelets can adjust their release of BDNF in response to multiple external factors that include anti-depressant medications [35], infection, and inflammation. Thus, plasma prepared to be platelet free may represent a more reliable measurement of steady state BDNF in the peripheral circulation. Another confounding factor for measuring peripheral BDNF levels in MCI is that BDNF has two distinct forms, pro- and mature. Notably, they have opposite effects, with pro-BDNF promoting neuronal cell death [36] while mature BDNF promotes neuronal cell growth and survival [19, 36]. However, many previous studies fail to differentiate between these two forms [25, 37]. Additionally, only two out of the six BDNF assays available selectively measured mature-BDNF, while the remaining assays combined the signals for both pro-BDNF and mature-BDNF [37]. This failure to differentiate between the forms of BDNF has been noted as another important factor skewing accurate measurements of BDNF in extant MCI studies, causing contradictory findings [25, 37].

To the best of our knowledge, the discriminative accuracies of plasma BDNF for MCI and different MCI subtypes have been largely unexamined. Apart from the neurotrophin hypothesis of cognitive impairment, with BDNF as one of the prominent biomarkers, there are inflammatory and stress hypotheses for the development of dementia [38, 39], suggesting that chronic low-grade inflammation and persistent stress increase the risk of developing cognitive impairment. In this regard, high-sensitivity C-reactive protein (hs-CRP) is a biomarker for low-grade chronic inflammation, whereas dehydroepiandrosterone sulfate (DHEA-S) is a biomarker for the hypothalamic–pituitary–adrenal axis (HPA), the primary physiological system regulating chronic stress. Although BDNF interacts with inflammatory and stress markers [40, 41], studies on plasma BDNF, hs-CRP and DHEA-S in MCI in the same study are scarce. This hypothesized neuro-immune-stress axis [42] could be evidenced by the presence of significant associations between peripheral BDNF, with hs-CRP and DHEA-S. Worthy of note is that, apart from MCI, these biomarkers have also been implicated in MDD and GAD [43–47]. In addition, adults with cerebral palsy (CP) whom often have co-morbid MCI, have similarly dysregulated biomarker levels [48], particularly similar hs-CRP level and lower plasma BDNF than MCI, hence making it even more imperative to control for the clinical and laboratory heterogeneity. Considering cases with co-morbid MDD, GAD, and CP will allow a deeper understanding of the utility of these biomarkers in discriminating MCI from cognitive healthy aging. In addition, associations between BDNF and cognitive function are rarely examined, hence investigating these associations will further our understanding on the roles of peripheral BDNF in modulating various cognitive functions impacted in MCI.

To address the gaps in knowledge outlined above, we compared two cohorts, healthy control (HC) and MCI, which had been controlled for the clinical and lab variability issues shared. This pilot study had four aims. First, we aimed to determine if the three biomarkers of interest, namely plasma BDNF, hs-CRP, and DHEA-S, have significantly different levels in participants with MCI compared to HC. Second, we aimed to determine the discriminative accuracies, sensitivities and specificities of these biomarkers in identifying MCI, in a series of sensitivity analyses that excluded those co-morbid with probable MDD, GAD, and CP [33]. Third, we investigated the associations, or the lack thereof, between: a) BDNF and hs-CRP/ DHEA-S, and b) the biomarkers and detailed neurocognitive tests. Lastly, as exploratory analyses, we separately investigated the levels and the discriminative accuracies of these three biomarkers in CP, probable MDD, and probable GAD, compared to HC.

## RESULTS

### Demographics

Table 1 summarizes the demographic characteristics of the study participants. We recruited a total of 160 participants, mean age=71.18, SD=5.66 (MCI cohort, n=40), mean age=66.95, SD=4.29 (HC cohort, n=56), and mean age=25, SD=5.39 (CP cohort, n=64; P<0.001). Most of the participants were female in both MCI and HC cohorts [MCI cohort n=28 (70%); HC cohort, n=45 (80.40%)], while relatively balanced sex in the CP cohort [n=29 (45.3%); <0.001]. Notably, the years of formal education differed significantly between the cohorts, as did the total number of chronic diseases and the proportion of cases with probable MDD and GAD. No significant differences were observed for BMI and plasma log DHEA-S.

**Table 1:** Demographic characteristics.

**Demographic characteristics**	**Mean ± SD *or* n (%)**			***P*-values**
	**MCI (n=40)**	**HC (n=56)**	**CP (n=64)**	
Age (in years)	71.18 ± 5.66	66.95 ± 4.29	25 ± 5.39	<0.001***
Sex				
Female	28 (70)	45 (80.40)	29 (45.3)	<0.001***
Male	12 (30)	11 (19.60)	35 (54.7)	
Years of formal education	4.33 ± 4.76	7.13 ± 3.66	13.45 ± 2.12	<0.001***
BMI	24.78 ± 4.13	24.03 ± 3.58	23.95 ± 5.19	0.634
Total number of chronic diseases	2.88 ± 1.53	1.91 ± 1.47	**-**	-
Plasma Log BDNF	3.19 ± 0.37	2.66 ± 0.27	2.07 ± 0.52	<0.001***
Plasma Log hs-CRP	0.21 ± 0.45	-0.06 ± 0.43	-0.05 ± 0.60	0.018*
Plasma Log DHEA-S	2.44 ± 0.52	2.39 ± 0.44	**-**	-
MCI subtypes- amnestic MCI	18 (45)	-	**-**	-
Non-amnestic MCI	22 (55)	-	**-**	-
Probable MDD-Yes	11 (27.5)	3 (5.4)	10 (15.9)	0.010*
No	29 (72.5)	53 (94.6)	53 (84.1)	
Probable GAD-Yes	5 (12.5)	5 (8.9)	28 (44.4)	<0.001***
No	35 (87.5)	51 (91.1)	35 (55.6)	

Notes: MCI, mild cognitive impairment; HC, healthy control; CP, cerebral palsy; n, Number; SD, Standard deviation; BMI, body-mass index; BDNF, Brain-derived neurotrophic factor; hs-CRP, High-sensitivity C-reactive Protein; DHEA-S, Dehydroepiandrosterone sulfate; NCA, neurocognitive tests; MDD, major depressive disorder; GAD, generalized anxiety disorder; NA, not applicable; * indicates p<0.05, ***indicates p<0.01, and ***indicates p<0.001; - = not available in the dataset.

### Plasma BDNF and hs-CRP, but not DHEA-S, were elevated in MCI versus HC

**Figure 1:**
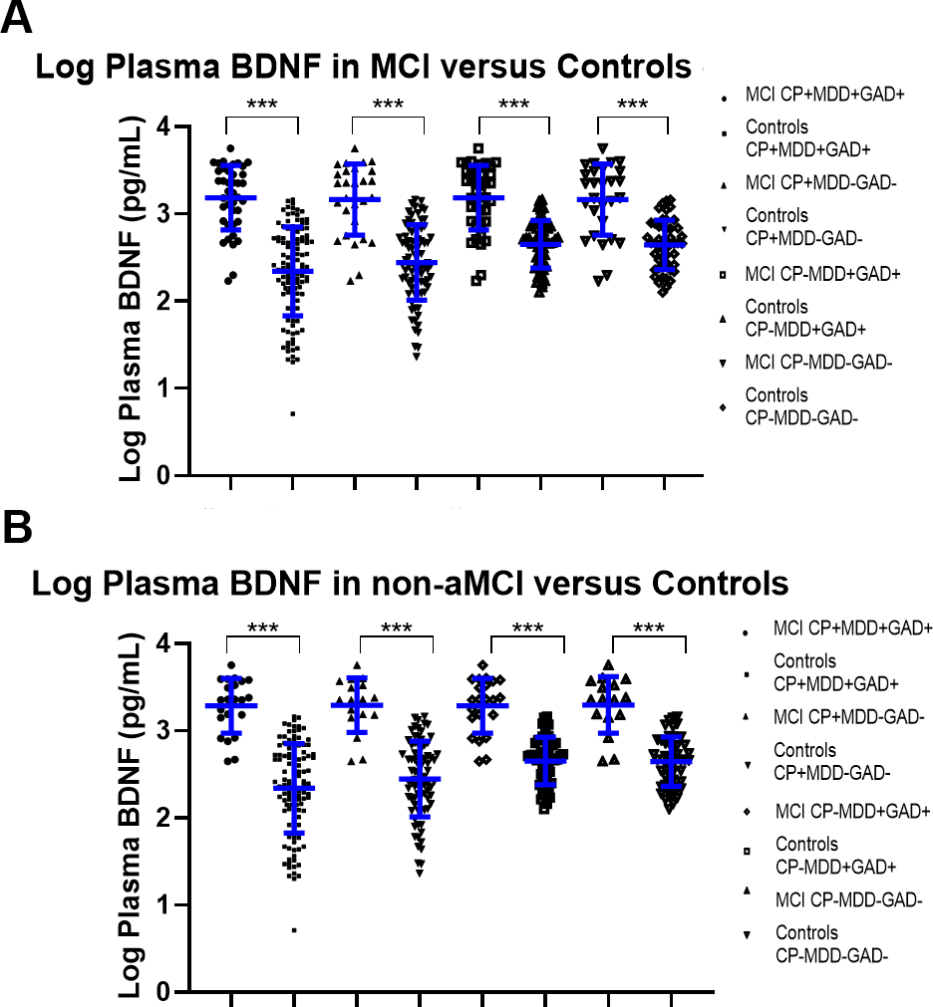
(**A**) Log Plasma BDNF levels in all-cause MCI cases compared to controls, with various sensitivity analyses including and removing co-morbidities. * indicates p<0.05, ***indicates p<0.01, and ***indicates p<0.001. (**B**) Log Plasma BDNF levels in non-amnestic (non-a) MCI cases compared to controls, with various sensitivity analyses including and removing co-morbidities.* indicates p<0.05, ***indicates p<0.01, and ***indicates p<0.001.

As shown in Figure 1A and Table 2A, plasma BDNF levels were significantly increased in MCI (n=40, 3.19±0.37 pg/mL), compared to HC (n=120, 2.34±0.51pg/mL, p<0.001). This effect remained once participants with CP, probable MDD and probable GAD were excluded from the analyses, resulting in an MCI population of (n=28, 3.16±0.40 pg/mL) and HC (n=48, 2.65±0.28pg/mL, p<0.001). Similarly, there were significantly increased plasma BDNF in both aMCI and non-aMCI (Table 2B, 2C). We further showed (Supplementary Table 1) that even after controlling for a range of other confounders, plasma BDNF remained significantly different in MCI compared to HC (β=0.47, 95% CI=0.32 to 0.62, p<0.001, R^2^=0.44).

**Table 2A:** Biomarker levels for each sub-group and their discriminative accuracies for all-cause MCI.

	**Inclusion of probable MDD and GAD?**	**Biomarkers**	**Biomarker levels (compared to control)**					**Discriminative accuracies**			
			**Clinically diagnosed MCI**		**Control**		**P-value**	**AUC**	**SE**	**95% CI of AUC**	**P-value**
			**n**	**Mean (SD)**	**n**	**Mean (SD)**					
CP cohort included	Included	Log BDNF	40	3.19 (0.37)	120	2.34 (0.51)	<0.001***	0.91	0.03	0.86 to 0.97	<0.001***
		Log hs-CRP	42	0.21 (0.44)	128	-0.05 (0.52)	0.004**	0.65	0.05	0.56 to 0.74	0.005**
	Excluded	Log BDNF	28	3.16 (0.41)	80	2.45 (0.43)	<0.001***	0.88	0.04	0.81 to 0.96	<0.001***
		Log hs-CRP	30	0.21 (0.44)	85	-0.07 (0.49)	0.008**	0.67	0.06	0.56 to 0.78	0.008**
CP cohort excluded	Included	Log BDNF	40	3.19 (0.37)	56	2.65 (0.27)	<0.001***	0.87	0.04	0.79 to 0.95	<0.001***
		Log hs-CRP	42	0.21 (0.44)	56	-0.06 (0.43)	0.003**	0.65	0.06	0.54 to 0.76	0.012*
		Log DHEA-S	42	2.46 (0.53)	56	2.39 (0.44)	0.440	0.50	0.06	0.37 to 0.62	0.947
	Excluded	Log BDNF	28	3.16 (0.40)	48	2.65 (0.28)	<0.001***	0.84	0.05	0.74 to 0.95	<0.001***
		Log hs-CRP	30	0.21 (0.44)	48	-0.08 (0.43)	0.006**	0.67	0.07	0.54 to 0.79	0.016*
		Log DHEA-S	30	2.41 (0.52)	48	2.42 (0.44)	0.897	0.43	0.07	0.29 to 0.57	0.322

**Table 2B:** Biomarker levels for each sub-group and their discriminative accuracies for non-aMCI.

	**Inclusion of probable MDD and GAD?**	**Biomarkers**	**Biomarker levels (compared to control)**					**Discriminative accuracies**			
			**Clinically diagnosed MCI**		**Control**		**P-value**	**AUC**	**SE**	**95% CI of AUC**	**P-value**
			**n**	**Mean (SD)**	**n**	**Mean (SD)**					
CP cohort included	Included	Log BDNF	22	3.29 (0.31)	120	2.34 (0.51)	<0.001***	0.95	0.02	0.91 to 1.00	<0.001***
		Log hs-CRP	24	0.16 (0.46)	128	-0.05 (0.52)	0.064	0.61	0.06	0.49 to 0.74	0.094
	Excluded	Log BDNF	16	3.29 (0.32)	80	2.45 (0.43)	<0.001***	0.94	0.03	0.88 to 1.00	<0.001***
		Log hs-CRP	18	0.21 (0.49)	85	-0.07 (0.49)	0.032	0.66	0.08	0.51 to 0.81	0.049*
CP cohort excluded	Included	Log BDNF	22	3.29 (0.31)	56	2.65 (0.27)	<0.001***	0.92	0.04	0.85 to 1.00	<0.001***
		Log hs-CRP	24	1.56 (0.46)	56	-0.06 (0.43)	0.046*	0.61	0.08	0.46 to 0.76	0.131
		Log DHEA-S	24	2.33 (0.53)	56	2.39 (0.44)	0.631	0.39	0.08	0.24 to 0.54	0.128
	Excluded	Log BDNF	16	3.29 (0.32)	48	2.65 (0.28)	<0.001***	0.92	0.04	0.84 to 1.00	<0.001***
		Log hs-CRP	18	0.21 (0.49)	48	-0.08 (0.43)	0.022*	0.65	0.09	0.48 to 0.82	0.067
		Log DHEA-S	18	2.34 (0.57)	48	2.42 (0.44)	0.554	0.35	0.09	0.18 to 0.52	0.067

Footnote: aMCI cases (n=18) were excluded from all the analyses presented in this table.

**Table 2C:** Biomarker levels for each sub-group and their discriminative accuracies for aMCI.

	**Inclusion of probable MDD and GAD?**	**Biomarkers**	**Biomarker levels (compared to control)**					**Discriminative accuracies**			
			**Clinically diagnosed MCI**		**Control**		**P-value**	**AUC**	**SE**	**95% CI of AUC**	**P-value**
			**n**	**Mean (SD)**	**n**	**Mean (SD)**					
CP cohort included	Included	Log BDNF	18	3.06 (0.41)	120	2.34 (0.51)	<0.001***	0.87	0.05	0.78 to 0.96	<0.001***
		Log hs-CRP	18	0.28 (0.41)	128	-0.05 (0.52)	0.011*	0.69	0.06	0.58 to 0.80	0.010*
	Excluded	Log BDNF	12	2.99 (0.45)	80	2.45 (0.43)	<0.001***	0.80	0.07	0.66 to 0.95	<0.001***
		Log hs-CRP	12	0.20 (0.37)	85	-0.07 (0.49)	0.077	0.68	0.07	0.55 to 0.82	0.041*
CP cohort excluded	Included	Log BDNF	18	3.06 (0.41)	56	2.65 (0.27)	<0.001***	0.80	0.07	0.66 to 0.94	<0.001***
		Log hs-CRP	18	0.28 (0.41)	56	-0.06 (0.43)	0.005**	0.70	0.07	0.57 to 0.83	0.011*
		Log DHEA-S	18	2.64 (0.49)	56	2.39 (0.44)	**0.044**	0.63	0.08	0.46 to 0.79	0.107
	Excluded	Log BDNF	12	2.99 (0.45)	48	2.65 (0.28)	0.026*	0.74	0.10	0.55 to 0.92	0.012*
		Log hs-CRP	12	0.20 (0.37)	48	-0.08 (0.43)	**0.045**	0.68	0.08	0.52 to 0.84	0.052
		Log DHEA-S	12	2.50 (0.44)	48	2.42 (0.44)	0.563	0.55	0.10	0.35 to 0.74	0.631

Footnote: non-aMCI cases (n=22) were excluded from all the analyses presented in this table.

Plasma hs-CRP had similar findings as BDNF. Hs-CRP had significantly increased levels for all-cause MCI and non-aMCI. Plasma DHEA-S did not have significant difference across all the models.

### High discriminative accuracies, sensitivities, and specificities of plasma BDNF for MCI and non-aMCI, including in sensitivity analyses removing cases with psychiatric co-morbidities

**Figure 2:**
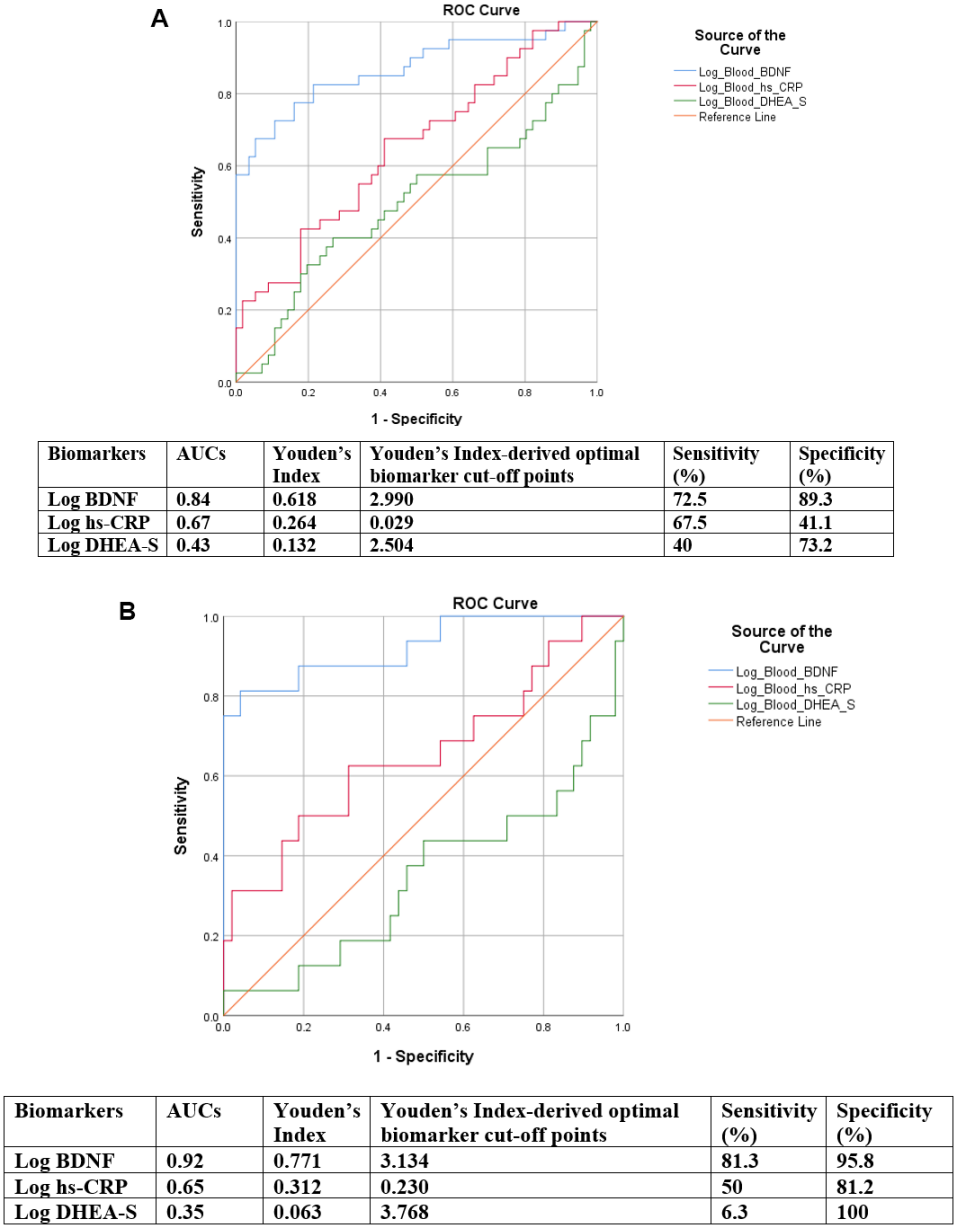
(**A**) ROC curves of discriminative accuracies of BDNF, hs-CRP and DHEA-S for all-cause MCI cases for the analysis removing all co-morbidities. (**B**) ROC curves of discriminative accuracies of BDNF, hs-CRP and DHEA-S for non-amnestic MCI for the analysis removing all co-morbidities.

As displayed in Table 2A, 2B and Figure 2A, 2B, upon removing cases with CP, probable MDD and GAD from the analyses, plasma BDNF showed good (AUC=0.84, 95% CI=0.74 to 0.95, p<0.001) and excellent discriminative accuracy (AUC=0.92, 95% CI=0.84 to 1.00, p<0.001) for all-cause MCI and non-amnestic MCI, respectively. Youden’s Index-derived optimal log BDNF cut-off point for all-cause MCI was 2.990, with sensitivity=72.5% and specificity=89.3%. Whereas for non-aMCI, the cut-off point was 3.134, with sensitivity=81.3% and specificity=95.8%. In addition, although the discriminative accuracy of BDNF for aMCI was comparatively lower, it still had good discriminative accuracy (AUC=0.74, 95% CI=0.55 to 0.92, p=0.012) (Table 2C).

### Associations between plasma BDNF and hs-CRP, but not with DHEA-S

To examine the presence of the proposed connections between the three physiological systems, we examined the associations between plasma BDNF and plasma hs-CRP or DHEA-S (Table 3). Bivariate association between plasma hs-CRP and BDNF were positively and significantly associated, with a higher level of plasma BDNF associated significantly with a higher level of plasma hs-CRP (β=0.27, 95%CI=0.05 to 0.49, p=0.018). However, the association was attenuated to become not significant (model 3: β=0.16, 95%CI= -0.07 to 0.39, p=0.168), when the covariate BMI was added. BDNF had no significant association with DHEA-S, even at the bivariate level, and this observation persisted through the subsequent models (p>0.05; Table 3).

**Table 3:** Associations between plasma BDNF with hs-CRP and DHEA-S.

**Biomarkers**	**Models**			
		**β (95% CI)**	***P* value**	***R^2^***
Log Hs-CRP	1	0.27 (0.05 to 0.49)	0.018*	0.06
	2	0.26 (0.02 to 0.50)	0.038*	0.07
	3^#^	0.16 (-0.07 to 0.39)	0.168	0.24
	4	0.15 (-0.08 to 0.38)	0.186	0.26
Log DHEA-S	1	0.07 (-0.18 to 0.31)	0.581	0.003
	2	0.04 (-0.13 to 0.21)	0.657	0.60
	3	0.03 (-0.14 to 0.21)	0.700	0.60
	4	0.01 (-0.16 to 0.19)	0.894	0.62

Footnotes: Independent variable in the model was Log BDNF, Brain-derived neurotrophic factor; dependent variables: Hs-CRP, High-sensitivity C-reactive Protein; DHEA-S, Dehydroepiandrosterone sulfate.

95% CI, 95% confidence interval; * indicates p<0.05; # indicates the significant covariate in model 3 was body-mass index (BMI).

Model 1: bivariate association.

Model 2: added age, sex, years of formal education.

Model 3: added body-mass index and the total number of chronic diseases.

Model 4: added two other biomarkers.

### Significant associations between plasma BDNF and multiple neurocognitive domains

In Table 4, we showed that higher plasma BDNF was significantly associated with lower cognitive test scores representing multiple cognitive domains, including Forward Digit Span (β=-1.17, 95%CI=-2.04 to -0.31, p=0.009), Backward Digit Span (β=-1.13, 95%CI= -1.83 to -0.42, p=0.002), Block Design Test (β= -4.86, 95%CI=-7.63 to -2.10, p=0.001), and Semantic Fluency (Animal Naming) Test (β=-1.76, 95%CI=-2.99 to -0.54, p=0.005). On the other hand, plasma BDNF was significantly associated with higher scores of Colour Trail Test I (β=23.77, 95%CI=5.70 to 41.85, p=0.011) and Colour Trail Test II (β=29.77, 95%CI=11.32 to 48.22, p=0.002). Notably, plasma BDNF alone accounted for 19-24% (R^2^=0.19 to 0.24) of the variance across different neurocognitive tests (model 1 of each respective test). On the contrary, plasma hs-CRP and DHEA-S had no significant associations with the neurocognitive tests (data not shown).

**Table 4:** Associations between plasma BDNF with multiple detailed neurocognitive tests.

**Detailed neurocognitive tests**	**Models**			
		**β (95% CI)**	***P-*value**	***R^2^***
Forward Digit Span	1	-1.86 (-2.66 to -1.05)	<0.001***	0.19
	2	-1.33 (-2.15 to -0.51)	0.002**	0.31
	3	-1.22 (-2.06 to -0.38)	0.005**	0.33
	4	-1.17 (-2.04 to -0.31)	0.009**	0.33
Backward Digit Span	1	-1.85 (-2.54 to -1.17)	<0.001***	0.24
	2	-1.24 (-1.91 to -0.56)	<0.001***	0.39
	3	-1.18 (-1.88 to -0.47)	0.001**	0.40
	4	-1.13 (-1.83 to -0.42)	0.002**	0.43
RAVLT Delayed Recall Test	1	-1.00 (-2.23 to 0.24)	0.11	0.03
	2	-0.36 (-1.57 to 0.86)	0.56	0.22
	3	-0.04 (-1.28 to 1.20)	0.95	0.26
	4	0.11 (-1.15 to 1.37)	0.87	0.28
RAVLT Delayed Recognition Test	1	-0.40 (-1.14 to 0.35)	0.29	0.01
	2	0.02 (-0.75 to 0.78)	0.97	0.16
	3	0.28 (-0.48 to 1.04)	0.47	0.23
	4	0.40 (-0.35 to 1.16)	0.29	0.28
Colour Trail Test I	1	45.18 (26.55 to 63.80)	<0.001***	0.20
	2	26.75 (9.36 to 44.13)	0.003**	0.43
	3	23.16 (5.37 to 40.96)	0.011*	0.45
	4	23.77 (5.70 to 41.85)	0.011*	0.47
Colour Trail Test II	1	48.95 (30.71 to 67.19)	<0.001***	0.24
	2	31.97 (14.53 to 49.40)	<0.001***	0.43
	3	30.46 (12.33 to 48.59)	0.001**	0.43
	4	29.77 (11.32 to 48.22)	0.002**	0.45
Block Design Test	1	-7.50 (-10.26 to -4.73)	<0.001***	0.24
	2	-5.40 (-8.05 to -2.75)	<0.001***	0.43
	3	-5.07 (-7.80 to -2.35)	<0.001***	0.45
	4	-4.86 (-7.63 to -2.10)	0.001**	0.46
Semantic Fluency (Animal Naming) Test	1	-3.19 (-4.37 to -2.00)	<0.001***	0.24
	2	-2.27 (-3.48 to -1.06)	<0.001***	0.35
	3	-2.03 (-3.27 to -0.80)	0.001**	0.38
	4	-1.76 (-2.99 to -0.54)	0.005**	0.42

Footnotes: Independent variable in the model was Log BDNF, Brain-derived neurotrophic factor; dependent variables, detailed neurocognitive tests; RAVLT, The Rey Auditory Verbal Learning Test.

95% CI, 95% confidence interval; * indicates p<0.05, ***indicates p<0.01, and ***indicates p<0.001.

Model 1: bivariate association.

Model 2: added age, sex, years of formal education.

Model 3: added body-mass index and the total number of chronic diseases.

Model 4: added two other biomarkers.

### Exploratory analyses - levels and discriminative accuracies of plasma BDNF, hs-CRP, and DHEA-S for CP, probable MDD, and probable GAD

For the co-morbidities, sensitivity analyses removing the co-morbidities showed a significantly higher level of hs-CRP in probable MDD (n=3, 0.44±0.16 ng/mL), compared to HC (n=53, -0.09±0.43 ng/mL, p=0.036) (Supplementary Table 2A), and borderline higher BDNF. DHEA-S seemed to be significantly lower in probable GAD cases (n=5, 2.01±0.31 ng/mL), compared to HC (n=51, 2.42±0.44 ng/mL, p=0.047) (Supplementary Table 2B). CP had significantly decreased plasma BDNF (Supplementary Table 2C) (n=32, 2.15±0.45 pg/mL, compared to HC (n=48, 2.65±0.28, p<0.001).

Shown in Table 2A, 2B and Figure 2A, 2B were discriminative accuracies of the three biomarkers for probable MDD, GAD, and CP separately (Supplementary Tables 2A–2C, respectively). Although significantly lower BDNF had great discriminative accuracies for CP (AUC=0.82, 95% CI=0.72 to 0.93, p=0.05), plasma BDNF had a comparatively much poorer discriminative accuracy for both MDD and GAD (AUC=0.62, 95% CI=0.49 to 0.75, p=0.478) and (AUC=0.47, 95% CI=0.22 to 0.72, p=0.829), respectively. Furthermore, elevated plasma hs-CRP had excellent discriminative accuracy for probable MDD (AUC=0.91, 95% CI=0.81 to 1.00, p=0.019) (Supplementary Table 2A).

## DISCUSSION

Addressing the clinical and laboratory heterogeneity causal of the contradictory evidence present in the literature, we found plasma BDNF to be significantly higher in MCI, compared to HC. Furthermore, elevated plasma BDNF had great discriminative accuracy for MCI, particularly excellent discriminative accuracy for non-aMCI. However, its discriminative accuracy for aMCI was poorer compared to those of total MCI and non-aMCI. These findings remained unchanged upon performing sensitivity analyses that excluded cases with co-morbid MDD and GAD, and sensitivity analyses with CP. Furthermore, regression models associating plasma BDNF and hs-CRP further support the presence of a neurotrophin-inflammation axis that is mainly modulated by BMI. Lastly, presence of significant associations with multiple neurocognitive tests also support BDNF as a modulator of several cognitive functions. Although plasma hs-CRP was significantly elevated in MCI, it had relatively poor discriminative accuracy, while DHEA-S did not show significant difference and had poor discriminative accuracy for MCI. Taken together, in the context of four neurological and psychiatric conditions, plasma BDNF, but not hs-CRP nor DHEA-S, appears to be a good and excellent biomarker to discriminate MCI and non-aMCI, respectively. Given plasma BDNF’s increased levels in MCI, along with its positive association with hs-CRP, and inverse associations with multiple neurocognitive domains, our data support the hypothesis that increased peripheral BDNF acts as a compensatory mechanism in the preclinical stage of dementia [14, 15].

Compared to HC, upon controlling for covariates, we found significantly higher plasma BDNF in MCI versus controls, regardless of whether we were assessing total or MCI sub-types. Furthermore, addressing four factors causing heterogeneity presents in previous studies [25, 28, 49], our pilot findings suggest that plasma BDNF had a high discriminative accuracy in differentiating MCI from HC. We also showed high discriminative accuracy, sensitivity and specificity for non-aMCI subtype, when more accurately measured peripheral BDNF was utilized. One previous study showed low and statistically insignificant discriminative accuracy of serum BDNF for MCI, which the author noted could have been caused by the high heterogeneity conferred by laboratory and clinical factors [50]. To our knowledge, only one previous study has examined the discriminative accuracy of plasma BDNF for aMCI. In that study, there was a lower level of plasma BDNF in aMCI compared to HC. We propose that controlling for the heterogeneity-causing variables addressed in this study explains the discrepancy in these findings. Given the encouraging findings presented in this study, plasma BDNF’s predictive accuracy for incident MCI, especially non-aMCI cases, should be examined in future longitudinal studies.

Although neuroinflammation is known to affect several BDNF-related signalling pathways [51], its relationship with plasma BDNF in MCI remains largely unknown. In this study, we found a significant association between plasma BDNF and plasma hs-CRP. Hence, our findings provide empirical evidence that increased BDNF levels appeared to be associated with chronic low-grade inflammation in MCI. However, the association diminished upon further controlling for BMI, which appeared as a significant covariate. This finding concurs with the central role of hs-CRP as a marker of obesity and metabolic syndrome [52, 53], suggesting that although there could a neuro-immune axis and thus cross-talks in preclinical dementia, it is heavily dependent on and modulated by BMI. This finding highlights the importance of controlling for BMI when examining hs-CRP and BDNF in MCI. Surprisingly, we detected no significant associations between BDNF with DHEA-S, the HPA-axis mediator even at the bivariate level. Another prominent biomarker for the HPA-axis is cortisol, which has been shown to be elevated in MCI [54, 55], but was not examined in this study. Future work will incorporate this as a possible biomarker candidate. In addition, changes in the HPA-axis and clinical presentations in MCI could instead be mediated by alterations in glucocorticoid receptor sensitivity and transcriptional regulators, including NFkB, or FKBP-5 [56]. Taken together, these findings corroborate the neurotrophic and inflammatory hypotheses for CI, with no support for a direct role of DHEA-S.

We showed increased plasma BDNF levels were significantly associated with worse cognition across multiple cognitive domains impacted in MCI. These findings concur with the expression of *BDNF* gene in various cortical areas corresponding to the cognitive functions associated with the detailed neurocognitive tests, including the cortex, hippocampus, and basal forebrain regions [17]. Furthermore, plasma BDNF alone accounted for 19-24% (R^2^=0.19 to 0.24) of the variance explained in each of the cognitive domains, providing further support for its potentially prominent functions in modulating cognition in preclinical dementia. Taken together with increased BDNF levels in MCI and its high discriminative accuracy, sensitivity and specificity, coupled with positive association with the inflammatory marker (hs-CRP), these inverse associations with neurocognitive tests further support the hypothesis of increased BDNF as a compensatory mechanism to counter neuronal insults at the MCI stage. We postulate that there might be a dementia stage-dependent function of plasma BDNF; At the MCI stage, although cognitive functions have generally deteriorated, plasma BDNF is upregulated as a compensatory mechanism and is thus associated with worse cognition. However, in the healthy aging and AD stages, BDNF likely serves a neuroprotective role and thus associates with better cognition, with higher peripheral BDNF levels protecting the older adults against dementia [19, 25]. On the contrary, plasma hs-CRP and DHEA-S had no significant associations with the neurocognitive tests. Future research needs to compare head-to-head plasma BDNF levels across the AD continuum and examine their differential associations with neurocognitive tests across different cognitive stages.

Lastly, as exploratory analyses, we also investigated the levels and the discriminative accuracies of the three biomarkers in three other co-morbid conditions and sensitivity analyses separately, namely probable MDD, probable GAD, and CP. CP had significantly lower plasma BDNF levels and high discriminative accuracy, compared to controls who were older adults without MCI. This finding concurs with our previous findings reporting decreased peripheral BDNF in CP compared to MCI [48]. In this study, we showed that after addressing the factors causing heterogeneity in plasma BDNF and removing cases with co-morbidities, the findings still hold, suggesting relative independence of plasma BDNF from psychiatric co-morbidities in CP. On the other hand, only when cases with co-morbidities were removed did hs-CRP appeared significantly lower with a higher discriminative accuracy for identifying cases with probable MDD. These findings might shed light on the contradictory findings on hs-CRP in MDD [45, 57, 58], as co-morbidities were frequently present in previous studies. Thus, these findings highlight the importance of considering co-morbidities when assessing hs-CRP in MDD. Due to the exploratory nature of the analyses for these three clinical conditions, future validations are necessary.

This study has several limitations. The main limitation was the relatively modest sample size and a non-matched case control study design. Future studies should validate these findings in larger independent cohorts. Second, although it was not within the scope of this pilot study, future studies should include neuroimaging techniques, such as amyloid positron emission tomography (PET), to pathologically confirm MCI diagnosis. Having said that, our participants were clinically diagnosed with MCI and characterized using a robust two-step clinical diagnostic procedure (assessor assessment followed by a consensus panel meeting). Third, while we understand that blood biomarkers may not be as accurate a reflection of brain pathophysiology as CSF, the core intention of this study was to examine plasma biomarkers, as potentially less invasive fluid biomarkers for screening for MCI. Fourth, several covariates were not available in the datasets, such as *BDNF* alleles, physical activity, social support, and homocysteine levels [19], leaving possible residual confounding. Our study also lacked a replication cohort. Thus, these encouraging pilot findings are preliminary and require further validation in other cohorts. It is worth noting that other similar studies with plasma BDNF measures in MCI and HC, which address all the concerns raised in this work, are not readily available to us. Finally, we did not have other diagnostic entities or canonical AD biomarkers to compare against BDNF. Due to the parsimonious involvements of BDNF in multiple psychiatric and neurological conditions, it is likely not a specific biomarker for a single condition, and perhaps more similar to neurofilament light (NfL) [59], is more likely a marker of neurodegeneration and neuroplasticity. Relatedly, previous works showed that despite having adequate statistical power, BDNF did not predict different neurodegenerative disease diagnostic statuses [60]. However, this may be again due to the lack of controlling for the confounders and variables we have discussed and addressed in this work.

In summary, this study addresses many well-established but often overlooked factors that cause high heterogeneity in BDNF levels. With that, we present this work as an important contribution to the body of knowledge supporting the compensatory roles of plasma BDNF in MCI, and show pilot data on the use of plasma BDNF to identify MCI from HC. In addition, by examining plasma BDNF in an Asian population, this study attempts to investigate the racial generalisability of current findings in the literature [25, 61], most of which are based primarily on Western-hemisphere populations. All participants were clinically well-characterised using well-established and validated instruments. Having multiple well-characterized clinical conditions in a single study conferred two main advantages: 1) we were able to perform multiple sensitivity analyses to mitigate the confounding effects of co-morbidities on the levels and discriminative accuracies of the biomarkers for MCI and therefore 2) we could provide pilot evidence comparing the levels and discriminative accuracies of the biomarkers in these four closely-related clinical conditions in a directly comparative manner. Further validation studies using longitudinal design and with larger sample sizes, as well as concurrently examining the levels of Aβ-42, p-Tau species, and NfL with plasma BDNF, could potentially support the use of plasma BDNF as a non-invasive biomarker for screening and triaging MCI diagnosis. Examinations of the relationship between BDNF in the cerebrospinal fluid (CSF) in future studies might also represent a promising avenue, allowing us to relate BDNF levels in plasma and CSF. These peripheral biomarkers might show negative correlations as is seen in increased salivary Aβ-42 [62, 63] versus decreased CSF Aβ-42 [64] in MCI and AD. Clarifying these relationships may further bolster the increased level and compensatory mechanism of plasma BDNF in preclinical dementia.

## MATERIALS AND METHODS

### Settings, study design, and participants

#### MCI cohort

This study [65, 66] was approved by the National University of Singapore ethics committee, Institutional Review Board (NUS-IRB Reference No: B-14-110), and registered with the clinical trial database (https://clinicaltrials.gov/ct2/show/NCT02286791).

The inclusion criterion was fulfilling the operational criteria of MCI based on The Diagnostic and Statistical Manual of Mental Disorders, Fifth Edition (DSM-V) [67]. We excluded older adults with either dementia or normal aging, had a neurological or major psychiatric condition, had a terminal illness, had visual or hearing impairments, had upper and lower limb motor difficulties, and those who were participating in another intervention at the time of the screening. Final diagnoses of MCI were made during the study’s consensus meetings chaired by a panel, consisting of at least two senior consultant-ranked psychiatrists, clinical scientists, and the trained assessors who administered the tests. Clinical diagnoses of cognitive status were made by adopting a robust two-step clinical diagnostic procedure (assessors’ initial assessments followed by a consensus panel meeting) in the primary psychiatry research and teaching hospital in Singapore. All included participants were psychotropic medication naïve.

#### Healthy control cohort

This study [68–71] was approved by the National University of Singapore Institutional Review Board (NUS IRB-Reference Code: B-15-016) and registered with clinicaltrials.gov, with the identifier: NCT02495194 (https://clinicaltrials.gov/ct2/show/NCT02495194).

The participants needed to be community-dwelling older adults between 60 and 85 years old. They needed to score a minimum of 22 points on the Montreal Cognitive Assessment (MoCA) and retained the ability to provide informed consent. Older adults with major psychiatric disorders and those with a medical history of stroke, epilepsy, ischemic heart disease, heart failure, chronic obstructive pulmonary disease, cancer, liver failure, and thyroid disorder were excluded. Older adults with marked upper and lower limb motor difficulties, significant visual or hearing impairment were also excluded. Lastly, those undergoing any concurrent interventions or therapies, including consumption of psychotropic medication(s), were also excluded. The same consensus panel for diagnosing MCI assessed the final psychiatric diagnoses of these participants.

#### Cerebral palsy cohort

The CP cross-sectional study [48, 72] was approved by the Colorado Multiple Institutional Review Board (COMIRB Reference No: 14-0367), and registered with the clinical trial database (https://clinicaltrials.gov/ct2/show/NCT02137005). The study was conducted at a clinical motion analysis laboratory at Children’s Hospital Colorado. The laboratory has a specialized team of clinicians and researchers and is internationally accredited by the Commission for Motion Laboratory Accreditation (CMLA) (http://www.cmlainc.org/).

This is a cross-sectional case-control study with the three cohorts analysed. For all three cohorts, only the baseline data were analysed in this study. Detailed descriptions of each cohort, including criteria for clinical and probable diagnoses, can be found in the Supplementary Materials and in the respective publications.

### Measures

#### Biomarker measurements

##### Blood sample collections

For all cohorts, blood collections were scheduled between 9:00 and 11:00 in the morning to minimize diurnal variations. The participants stopped consumption of foods after 10 pm the night before venepuncture. The consumption of only water was advised. To reduce the confounding effects of stressors on the biomarkers, the participants were advised not to exercise or perform rigorous physical activities before the collections and not to rush to the research center if running late. Blood draw via venepuncture was performed by the research nurses on the day that the participants visited the research center. The blood samples were collected in K2-EDTA spray coated blood vacutainers (BD, New Jersey, USA). The blood samples were kept at 4° C for a maximum of three hours before being processed in the respective laboratories.

#### Enzyme-linked immunosorbent assay (ELISA) measurements of plasma biomarkers

To obtain plasma upon samples arriving at the lab, whole blood samples were centrifuged at 1650g for 25 min at room temperature. Subsequently, plasma samples were bio-banked at −80° C until study completion, after which all the samples were assayed in one batch. We employed commercially available ELISA kits to quantify the level of three plasma biomarkers, namely BDNF (Promega Corporation, Madison, WI, USA), hs-CRP (Tecan, Männedorf, Switzerland), and DHEA-S (CUSABIO, Houston, TX, USA). All experiments were performed as per instructions of respective manufacturers of the kits. For quantifying only the mature form of BDNF, we adhered to the protocol’s instructions in pre-processing the samples before running ELISA. Specifically, to measure total BDNF using this assay, an acid treatment to the plasma sample is typically performed. However, for the purpose of this study to only measure free mature BDNF, the acid treatment procedures were skipped. Coefficient variations (CVs) of all the assays were <10%. All personnel who processed the samples and ran the ELISA were blinded to the diagnostic status of the participants.

#### Neurocognitive tests

Cognitive functions were examined using neurocognitive tests that have been validated to have good content validity and psychometric properties internationally and in the local context [73]. Details of the tests can be found in Supplementary Table 3. Higher scores in all neurocognitive tests indicate higher cognitive functions, except for the colour trail tests I and II, where lower test scores indicate higher cognitive functions.

### Statistical analyses

Informed by our previous systematic review and meta-analysis on this topic [25], a cohort size of 60 is capable of detecting statistically significant difference in peripheral BDNF levels, assuming a 20% difference, with a power of 0.80 and type-I error of 0.05 [74]. All measures were expressed as mean ± standard deviation (SD) for continuous measures and as number (percentage) for categorical measures. The differences in variables were examined using Student’s *t*-test, chi-square or Fisher’s exact tests, as the data necessitated. The raw values of the biomarker measurements did not fulfil the normality assumption; therefore, the raw values of the biomarkers were log-transformed for subsequent analyses and were successfully normalized, based on dot plots, skewness, and kurtosis. On the other hand, the raw values of the detailed neurocognitive tests conformed to statistical normality and thus were not transformed. To address aim 1, we performed two sets of analyses: one without controlling for covariates and another using linear regression analyses. In linear regression analyses, using a bivariate model, we first associated a dummy variable representing MCI or HC cohort (independent variable) with biomarkers (dependent variables) separately. Subsequently, we controlled for covariates, including age, gender, and years of formal education in model 2 and further controlled for additional covariates, namely body-mass index (BMI) and the total number of chronic diseases in model 3. To investigate aim 2, we determined the discriminative accuracies of these biomarkers for MCI by employing concordance (C-) statistics, with area under the curve (AUC) values as indicators. An AUC of ≥ 0.9 was considered excellent, ≥ 0.8 considered good, and ≥ 0.7 considered fair [75]. Attempting to disentangle the confounding effects of the other three neurological and psychiatric conditions, namely CP, probable MDD, and GAD, on the discriminative accuracies of biomarkers, we performed sensitivity analyses where: 1) all receiver operating characteristic curve (ROCs) were run excluding the CP cohort, and 2) all analyses were run excluding all cases with probable MDD and GAD and 3) permutations of the two. Additionally, for biomarkers with high AUCs for MCI, the Youden index [*J*] was calculated balancing sensitivity and specificity, which provides indications on the performance of a biomarker at an optimal cut-off point. The Youden index (*J* = sensitivity + specificity - 1) has a maximum value of 1 (indicating a perfect test) and a minimum of 0, which is when the test has no diagnostic value. Hence, a useful biomarker should have a Youden Index exceeding 0.5, and the higher the value the better [76]. In Figure 2A, 2B, we reported the Youden’s Index, the index-derived optimal biomarker cut-off points, specificities, and sensitivities. To investigate aim 3(a), we ran linear regressions associating BDNF with hs-CRP/DHEA-S, using stepwise regression models with the same covariates indicated previously included in the models, with the additional model 4 further controlled for two other biomarkers. To examine aim 3(b), we ran a separate set of linear regression models to determine if there were significant associations between the biomarkers with the detailed neurocognitive tests, sequentially controlling for covariates. To examine aim 4, similar ROC analyses as were carried out for addressing aim 2 were performed. All analyses were performed using the Statistical Package for the Social Sciences (SPSS) Statistics for Windows, version 24.0 (IBM Corp., Armonk, NY, USA). Missing values were excluded case-wise for all analyses. ROC curves and dot plots were generated using SPSS and GraphPad Prism version 6 (GraphPad Software, San Diego, CA, USA), respectively. A two-tailed p-value of <0·05 was considered statistically significant. Due to the pilot nature of this study, we did not control for multiple testing [77, 78].

## Supplementary Materials

Supplementary Materials

Supplementary Tables
