# Supplementary material for: Increased plasma brain-derived neurotrophic factor (BDNF) as a potential biomarker for and compensatory mechanism in mild cognitive impairment: a case-control study
Source: Aging (Albany NY). 2021 Oct 15;13(19):22666–89. doi: 10.18632/aging.203598 (PMC8544315; doi:10.18632/aging.203598)
Supplement: Supplementary Materials [file aging-13-203598-s001.pdf]

SUPPLEMENTARY MATERIALS

Supplementary Methods

CP cohort inclusion and exclusion criteria [1–6]

Participants were included in the study if they were (1) interested and able to participate in the study and (2) able to walk across a 35-foot (10.6m) walkway, with or without assistive devices, at least three times.

Determining cases with probable MDD and GAD across different cohorts

For all cases of probable major depressive disorder (MDD) and probable generalized anxiety disorder (GAD), we used the respective cut-off points for each of the scales employed in the respective cohorts. Each cohort has different scales and hence we employed the scales' respective established cut-offs for establishing probable cases.

MCI cohort

GDS

The 15-item Geriatric Depression Scales (GDS) was used to assess depressive symptoms. The participants rated 15 items with either Yes (1) or No (0) response categories. The possible scores range from zero to 15, with a higher score indicating a higher depressive symptom. The cut-off for probable MDD was established as GDS≥5 [7].

GAI

The 20-item Geriatric Anxiety Inventory (GAI) was used to assess anxiety symptoms [8]. The participants rated the items with either agree (1) or disagree (0). The possible scores range from zero to 20, with a higher score indicating a higher anxiety symptom. The cut-off for probable GAD was established as GAI≥9 [8].

HC cohort

For both the Zung's instruments, they are 20-item assessments of depressive and anxiety symptoms. Participants rated each item depending on how they felt during the past week using a 4-point likert scale ranging from 1 to 4. A higher score indicated more severe symptoms. The cut-offs were SDS≥42 and SAS≥36 based on previous studies [9, 10].

CP cohort

CES-D

A higher total score indicated a higher number of depressive symptoms. The cut-off was set at ≥16 [11]. Furthermore, using the Structured Clinical Interview for Diagnostic and Statistical Manual of Mental Disorders, Revised Third Edition (SCID), the "gold standard" clinical tool for diagnosing depression, CES-D has high sensitivity (92%-100%) and specificity (84%-87%) in correctly classifying cases with depression [12, 13].

PROMIS emotional distress—anxiety— short form

Subjects were asked questionnaire to assess anxiety symptoms during the past 7 days on a 5-point likert scale ranging from 0-5. A higher score indicated a higher anxiety symptoms. The raw scores were then converted into T-scores. A cut-off of T-scores ≥55 indicated some level of clinically-significant anxiety [14].

Supplementary References

1. Baer HR, Thomas SP, Pan Z, Tagawa A, Carollo JJ, Heyn PC. Self-reported physical function is associated with walking speed in adults with cerebral palsy. J Pediatr Rehabil Med. 2019; 12:181–88. https://doi.org/10.3233/PRM-180585 PMID:31227669

2. Carollo J, Tagawa A, Bodkin A, Pan Z, Heyn PC. Analysis of walking in individuals with CP during transition to adulthood: From The Cpat Study. Arch Phys Med Rehabil. 2018; 99: e72. https://doi.org/10.1016/j.apmr.2018.07.254

3. Heyn P, Robertson D, Pan Z, Carollo J. Examination of premature aging in individuals with cerebral palsy by self-reported health status. The Gerontologist. 2016; 56:471. https://doi.org/10.1093/geront/gnw162.1890

4. Heyn P, Tagawa A, Pan Z, Bodkin A, Carollo J. Association Between Gait Speed and Cognitive Function in Adults with Cerebral Palsy. Arch Phys Med Rehabil. 2017; 98: e35–6.

5. Heyn PC, Tagawa A, Pan Z, Thomas S, Carollo JJ. Prevalence of metabolic syndrome and cardiovascular disease risk factors in adults with cerebral palsy. Dev Med Child Neurol. 2019; 61:477–83. https://doi.org/10.1111/dmcn.14148 PMID:30663044

6. Oliveira T, Carollo J, Robertson D, Pan Z, Heyn P. Incidence of Epilepsy in Adults with Cerebral Palsy and Secondary Health Outcomes: A Review and Proposed Feasibility Study. J Neurol Disord. 2014; 2: 2.

7. Yesavage JA, Brink TL, Rose TL, Lum O, Huang V, Adey M, Leirer VO. Development and validation of a geriatric depression screening scale: a preliminary report. J Psychiatr Res. 1982; 17:37–49.

https://doi.org/10.1016/0022-3956(82)90033-4 PMID:7183759

8. Pachana NA, Byrne GJ, Siddle H, Koloski N, Harley E, Arnold E. Development and validation of the Geriatric Anxiety Inventory. Int Psychogeriatr. 2007; 19:103–14. https://doi.org/10.1017/S1041610206003504 PMID:16805925

9. Dunstan DA, Scott N. Clarification of the cut-off score for Zung's self-rating depression scale. BMC Psychiatry. 2019; 19:177. https://doi.org/10.1186/s12888-019-2161-0 PMID:31185948

10. Dunstan DA, Scott N. Norms for Zung's Self-rating Anxiety Scale. BMC Psychiatry. 2020; 20:90. https://doi.org/10.1186/s12888-019-2427-6 PMID:32111187

11. Vilagut G, Forero CG, Barbaglia G, Alonso J. Screening for Depression in the General Population with the Center for Epidemiologic Studies Depression (CES-D): A Systematic Review with Meta-Analysis. PLoS One. 2016; 11:e0155431. https://doi.org/10.1371/journal.pone.0155431 PMID:27182821

12. Spitzer RL, Williams JB, Gibbon M, First MB. The Structured Clinical Interview for DSM-III-R (SCID). I: History, rationale, and description. Arch Gen Psychiatry. 1992; 49:624–29. https://doi.org/10.1001/archpsyc.1992.01820080032005 PMID:1637252

13. Lyness JM, Noel TK, Cox C, King DA, Conwell Y, Caine ED. Screening for depression in elderly primary care patients. A comparison of the Center for Epidemiologic Studies-Depression Scale and the Geriatric Depression Scale. Arch Intern Med. 1997; 157:449–54. PMID:9046897

14. American Psychiatric Association. Diagnostic and statistical manual of mental disorders (DSM-5®). American Psychiatric Pub. 2013.
