# Supplementary material for: Increased plasma brain-derived neurotrophic factor (BDNF) as a potential biomarker for and compensatory mechanism in mild cognitive impairment: a case-control study
Source: Aging (Albany NY). 2021 Oct 15;13(19):22666–89. doi: 10.18632/aging.203598 (PMC8544315; doi:10.18632/aging.203598)
Supplement: Supplementary Tables [file aging-13-203598-s002.pdf]

SUPPLEMENTARY TABLES

Supplementary Table 1. Linear regressions between biomarkers (dependent variables) separately with dummy variable indicating cohort (and diagnostic) status, i.e. MCI and HC (independent variable).

Biomarkers	Models	β (95% CI)	*P* value	R^2
	1	0.53 (0.40 to 0.66)	**<0.001*** **	0.41
BDNF	2	0.48 (0.34 to 0.63)	**<0.001*** **	0.43
	3	0.47 (0.32 to 0.62)	**<0.001*** **	0.44
	1	0.27 (0.09 to 0.44)	**0.004** **	0.09
Hs-CRP	2	0.27 (0.07 to 0.47)	**0.009** **	0.10
	3	0.22 (0.03 to 0.41)	**0.027***	0.26
	1	0.08 (-0.12 to 0.27)	0.45	0.006
DHEA-S	2	0.06 (-0.08 to 0.20)	0.42	0.60
	3	0.06 (-0.08 to 0.21)	0.39	0.61

Notes: BDNF, Brain-derived neurotrophic factor; Hs-CRP, High-sensitivity C-reactive Protein; DHEA-S, Dehydroepiandrosterone sulfate.
95% CI=95% confidence interval, * indicates p<0.05, ***indicates p<0.01, and ***indicates p<0.001.
Model 1: bivariate association.
Model 2: added age, sex, years of formal education.
Model 3: added body-mass index and the total number of chronic diseases.

Supplementary Table 2A. Biomarker levels for each analysis and their discriminative accuracies for probable MDD.

	Inclusion of clinically-diagnosed MCI?	Biomarkers*	Biomarker levels (compared to control)					Discriminative accuracies			
			Probable MDD		Control		P-value	AUC	SE	95% CI of AUC	P-value
			n	Mean (SD)	n	Mean (SD)					
CP cohort included	Included	BDNF	24	2.75 (0.70)	135	2.53 (0.57)	0.096	**0.64**	**0.06**	**0.51 to 0.76**	**0.032***
		**hs-CRP**	**26**	**20.25 (0.47)**	**143**	**-0.04 (0.51)**	**0.009** **	**0.69**	**0.06**	**0.58 to 0.80**	**0.003** **
	Excluded	BDNF	13	2.31 (0.64)	106	2.36 (0.49)	0.753	0.53	0.08	0.36 to 0.69	0.772
		**hs-CRP**	**15**	**0.23 (0.51)**	**112**	**-0.09 (0.51)**	**0.020** **	**0.74**	**0.08**	**0.58 to 0.89**	**0.005** **
CP cohort excluded	Included	**BDNF**	**14**	**3.15 (0.33)**	**82**	**2.83 (0.41)**	**0.006** **	**0.74**	**0.06**	**0.62 to 0.85**	**0.005** **
		**hs-CRP**	**14**	**0.30 (0.40)**	**84**	**0.01 (0.45)**	**0.025***	**0.69**	**0.07**	**0.55 to 0.83**	**0.024***
		DHEA-S	14	2.60 (0.52)	84	2.39 (0.47)	0.126	0.62	0.09	0.45 to 0.80	0.152
	Excluded	**BDNF**	**3**	**2.73 (0.02)**	**53**	**2.65 (0.28)**	**0.048***	0.62	0.07	0.49 to 0.75	0.478
		**hs-CRP**	**3**	**0.44 (0.16)**	**53**	**-0.09 (0.43)**	**0.036***	**0.91**	**0.05**	**0.81 to 1.00**	**0.019***
		DHEA-S	3	2.44 (0.49)	53	2.38 (0.45)	0.840	0.53	0.16	0.22 to 0.83	0.870

Supplementary Table 2B. Biomarker levels for each analysis and their discriminative accuracies for probable GAD.

	Inclusion of clinically-diagnosed MCI?	Biomarkers	Biomarker levels (compared to control)					Discriminative accuracies			
			Probable GAI		Control		P-value	AUC	SE	95% CI of AUC	P-value
			n	Mean (SD)	n	Mean (SD)					
CP cohort included	Included	**BDNF**	**38**	**2.25 (0.70)**	**121**	**2.65 (0.54)**	**0.002****	**0.67**	**0.06**	**0.56 to 0.78**	**0.002****
		hs-CRP	40	-0.07 (0.58)	129	0.03 (0.49)	0.260	0.55	0.06	0.44 to 0.66	0.323
	Excluded	**BDNF**	**33**	**2.09 (0.60)**	**86**	**2.44 (0.44)**	**0.003****	**0.68**	**0.06**	**0.56 to 0.79**	**0.003****
		hs-CRP	35	-0.11 (0.57)	92	-0.04 (0.50)	0.473	0.53	0.06	0.41 to 0.65	0.650
CP cohort excluded	Included	BDNF	10	2.99 (0.42)	86	2.86 (0.41)	0.370	0.41	0.09	0.23 to 0.59	0.337
		hs-CRP	10	0.01 (0.54)	88	0.06 (0.44)	0.727	0.58	0.11	0.37 to 0.79	0.401
		DHEA-S	10	2.43 (0.57)	88	2.42 (0.47)	0.926	0.48	0.11	0.28 to 0.69	0.857
	Excluded	BDNF	5	2. 66 (0.27)	51	2.65 (0.28)	0.929	0.47	0.13	0.22 to 0.72	0.829
		hs-CRP	5	-0.19 (0.39)	51	-0.05 (0.43)	0.468	0.65	0.12	0.41 to 0.89	0.269
		**DHEA-S**	**5**	**2.01 (0.31)**	**51**	**2.42 (0.44)**	**0.047***	0.75	0.10	0.56 to 0.95	0.064

Footnote: in contrast to MCI and probable MDD (higher values), AUC curves were generated with the lower values of the biomarkers discriminating probable GAD from other conditions.

Supplementary Table 2C. Biomarker levels for each analysis and their discriminative accuracies for CP.

	Inclusion of probable MDD and GAD?	Biomarkers	Biomarker levels (compared to control)					Discriminative accuracies			
			Clinically diagnosed CP		Control		P-value	AUC	SE	95% CI of AUC	P-value
			n	Mean (SD)	n	Mean (SD)					
MCI cohort included	Included	**BDNF**	**64**	**2.07 (0.52)**	**96**	**2.87 (0.04)**	**<0.001*****	**0.88**	**0.03**	**0.83 to 0.94**	**<0.001*****
		hs-CRP	72	-0.05 (0.58)	98	0.05 (0.45)	0.210	0.57	0.05	0.48 to 0.67	0.130
	Excluded	**BDNF**	**32**	**2.15 (0.45)**	**76**	**2.84 (0.42)**	**<0.001*****	**0.87**	**0.04**	**0.79 to 0.95**	**<0.001*****
		hs-CRP	37	-0.05 (0.57)	78	0.03 (0.45)	0.397	0.59	0.07	0.46 to 0.72	0.150
MCI cohort excluded	Included	**BDNF**	**64**	**2.07 (0.52)**	**56**	**2.65 (0.27)**	**<0.001*****	**0.83**	**0.04**	**0.76 to 0.91**	**<0.001*****
		hs-CRP	72	-0.05 (0.58)	56	-0.06 (0.43)	0.902	0.52	0.05	0.41 to 0.62	0.740
	Excluded	**BDNF**	**32**	**2.15 (0.45)**	**48**	**2.65 (0.28)**	**<0.001*****	**0.82**	**0.05**	**0.72 to 0.93**	**<0.001*****
		hs-CRP	37	-0.05 (0.57)	48	-0.08 (0.43)	0.820	0.54	0.07	0.40 to 0.68	0.549

Footnote: in contrast to MCI and probable MDD (higher values for discriminating the conditions) and similar to probable GAD, AUC curves were generated with the lower values of the biomarkers discriminating CP from other conditions. DHEA-S was not examined in the CP cohort and hence was not presented in this table.

Supplementary Table 3. Neurocognitive test and its associated cognitive domain(s) and task description.

Neurocognitive test	Cognitive domain(s)	Task description
RAVLT [1]	Declarative verbal learning and memory (immediate, delayed, and recognition)	Participants were given a list of 15 unrelated words (list A) to learn and immediately recall aloud over five learning trials (Immediate Recall). Subsequently, an interference list of 15 unrelated words (list B) was presented only once for the participants to learn and recall immediately. After which, participants were instructed to recall aloud the words from list A. Approximately 30 minutes later, they were again asked to recall aloud the words from list A (Delayed Recall). Finally, participants were given a list of 50 words, comprising list A, list B, and 20 new distractor words, from which they had to identify the original 15 words (Recognition). Eight outcome measures were used in RAVLT. RAVLT T1 and RAVLT T5 referred to the total number of words correctly recalled in the first and fifth learning trials from list A during Immediate Recall. RAVLT B referred to the total number of words correctly recalled from the interference list. RAVLT T6 referred to the total number of words correctly recalled from list A during Delayed Recall. Lastly, RAVLT Recognition Trial and RAVLT Recognition Trail – False Positive referred to the total number of words correctly identified and falsely identified from list A during Recognition.
Digit Span Forward and Backward Task	Attention and working memory	The Digit Span Forward and Backward Task are subtests from the Wechsler Adult Intelligence Scale III (WAIS-III) [2]. A series of numbers were read aloud by the assessor, of which participants were required to repeat the series of numbers in the same (forward) or reverse (backward) order. The forward trial measures working memory span specifically, while the backward trial involved manipulation of information in the working memory. Two outcome measures were used in the Digit Span Task. Forward and Backward scores were obtained from the total number of forward and backward trials successfully repeated by the participants, respectively.
CTT [3]	Divided attention	The CTT consists of two parts. In the first (CTT1), participants connected a series of numbers that were printed within pink and yellow circles, sequentially from 1 to 25. In the second part (CTT2), participants similarly connected the numbers from 1 to 25, but alternated between choosing numbers in either pink or yellow circles. Three outcome measures were obtained from CTT – completion time for CTT1 and CTT2, and interference effect (i.e. CTT interference), which was calculated as the difference in completion times between CTT1 and CTT2, divided by CTT1.
Block Design Test	Visuospatial function	The Block Design Test is a subtest from the WAIS-III [2]. Here, participants were instructed to arrange blocks with red and white patterns on different sides to match the required block patterns in each trial. Scoring for the block design test depended on both the accuracy in matching the patterns and speed. Additional points were awarded to participants if they completed the trials within various time limits.
Semantic Fluency (Animal) Test [4]	Verbal fluency	Participants were instructed to name as many different animals as they could in one minute. The total score was indicated by the total number of correct and unique animal names.

Notes: RAVLT, Rey Auditory Verbal Learning Test; CTT, Color Trails Test.

REFERENCES

1. Rey A. L’examen psychologique dans les cas d’encéphalopathie traumatique.(Les problems.). Arch Psychol. Editions Médecine et Hygiène. 1941.
2. Wechsler D. WAiS-iii. Psychological Corporation San Antonio, TX. 1997.
3. D’Elia L, Satz P, Uchiyama C, White T. Psychological Assessment Resources. Inc California, 1994.
4. Lezak MD, Howieson DB, Loring DW, Fischer JS. Neuropsychological assessment. Oxford University Press, USA. 2004.
